# Nutraceutical Potential of Phenolics from ′Brava′ and ′Mansa′ Extra-Virgin Olive Oils on the Inhibition of Enzymes Associated to Neurodegenerative Disorders in Comparison with Those of ′Picual′ and ′Cornicabra′

**DOI:** 10.3390/molecules23040722

**Published:** 2018-03-21

**Authors:** María Figueiredo-González, Patricia Reboredo-Rodríguez, Carmen González-Barreiro, Alegría Carrasco-Pancorbo, Jesús Simal-Gándara, Beatriz Cancho-Grande

**Affiliations:** 1 Nutrition and Bromatology Group, Department of Analytical and Food Chemistry, Faculty of Food Science and Technology, University of Vigo, Ourense Campus, E-32004 Orense, Spain; mariafigueiredo@uvigo.es(M.F.-G.); preboredo@uvigo.es (P.R.-R.); cargb@uvigo.es (C.G.-B.); jsimal@uvigo.es (J.S.-G.); bcancho@uvigo.es (B.C.-G.); 2 Dipartimento di Scienze Cliniche Specialistiche ed Odontostomatologiche (DISCO)-Sez. Biochimica, Facoltà di Medicina, Università Politecnica delle Marche, Via Ranieri 65, 60131 Ancona, Italy; 3 Department of Analytical Chemistry, Faculty of Science, University of Granada, Ave. Fuentenueva s/n, 18071 Granada, Spain; alegriac@ugr.es

**Keywords:** extra-virgin olive oil, cholinesterases, monoamine oxidases, lipoxygenase, phenolic compounds, neurodegenerative disorders, Alzheimer′s disease, Parkinson′s disease, major depressive disorder

## Abstract

The increasing interest in the Mediterranean diet is based on the protective effects against several diseases, including neurodegenerative disorders. Polyphenol-rich functional foods have been proposed to be unique supplementary and nutraceutical treatments for these disorders. Extra-virgin olive oils (EVOOs) obtained from ′Brava′ and ′Mansa′, varieties recently identified from Galicia (northwestern Spain), were selected for in vitro screening to evaluate their capacity to inhibit key enzymes involved in Alzheimer′s disease (AD) (acetylcholinesterase (AChE), butyrylcholinesterase (BuChE) and 5-lipoxygenase (5-LOX)), major depressive disorder (MDD) and Parkinson′s disease (PD) (monoamine oxidases: *h*MAO-A and *h*MAO-B respectively). ′Brava′ oil exhibited the best inhibitory activity against all enzymes, when they are compared to ′Mansa′ oil: BuChE (IC_50_ = 245 ± 5 and 591 ± 23 mg·mL^−1^), 5-LOX (IC_50_ = 45 ± 7 and 106 ± 14 mg·mL^−1^), *h*MAO-A (IC_50_ = 30 ± 1 and 72 ± 10 mg·mL^−1^) and *h*MAO-B (IC_50_ = 191 ± 8 and 208 ± 14 mg·mL^−1^), respectively. The inhibitory capacity of the phenolic extracts could be associated with the content of secoiridoids, lignans and phenolic acids.

## 1. Introduction 

The recognized beneficial properties of plant polyphenols are used in the design of drugs as neuroactive multitarget directed ligands (MTDLs) designed to combat the progression of major depressive disorder (MDD), and of neurodegenerative disorders, such as Alzheimer′s (AD) and Parkinson′s (PD) diseases, between others [1]. There is substantial evidence that indicates that polyphenols exert diverse roles in the central nervous system (CNS), through multiple mechanisms as antioxidants, antiamyloidogenic and anti-inflammatories. Also, the neuroprotective effects of polyphenols involve mainly signaling pathway mediators, inhibition of neurotoxicity, and modulation of enzymes in neurotransmission [2,3,4,5,6]. 

Regarding the last mechanism, it is known that reduced levels of the neurotransmitters in the synaptic cleft are engaged in the pathophysiology of several CNS disorders. Two major cholinesterase (ChEs), acetylcholinesterase (AChE) and butyrylcholinesterase (BuChE) play an important role in AD. Based on the cholinergic hypothesis, high levels of these enzymes reduce the levels of the neurotransmitter acetylcholine (ACh) in the synaptic gap, consequently, an increase of the AD progression occurs. Therefore, restoring the levels of the neurotransmitter acetylcholine (ACh) neurotransmitter via inhibition of AChE and BuChE enzymes is actually the most useful therapeutic approach to treat AD and other forms of dementia [7,8].

Monoamine oxidase isoforms (*h*MAOs) are located on the outer mitochondrial membrane and are present in all type of cells at different proportions and concentrations. *h*MAO-A and *h*MAO-B catalyze the oxidative deamination of a variety of neurotransmitters. *h*MAO-A inhibitors are used as antidepressant agents for MDD with a central serotonin and noradrenaline deficiency and *h*MAO-B inhibitors are applied in PD therapy, where a central dopamine deficiency is responsible for the characteristic motor deficits [9,10].

Additionally, the neuroinflammation is also closely related to the pathogenesis of AD and PD. 5-lipoxygenase (5-LOX) enzyme has an important role in inflammation catalyzing the conversion of arachidonic acid released from membranes, into an oxidized compound, which is further metabolized into different leukotrienes considered as potent inflammatory mediators. Thus, 5-LOX inhibitors could effectively reduce inflammation associated with neurodegenerative disorders [11,12,13]. 

Some phenolic compounds have already addressed to exhibit several remarkable biological activities on inhibition of the previously described enzymes: *p*-coumaric acid, luteolin and lignans including pinoresinol and syringaresinol as owners of anticholinesterase activities [14,15,16,17]; oleocanthal, hydroxytyrosol, *p*-coumaric acid, and luteolin with inhibitory activity against 5-LOX [18,19,20] and flavonoids including apigenin and luteolin as responsible of the inhibition of *h-*MAOs [21,22]. 

These phenolic compounds are found in the virgin olive oil (EVOO), the major source of energy in the Mediterranean diet. Recent epidemiological evidence and clinical trials connect the Mediterranean diet (Med Diet) with protective effects against the health. The latest reports have highlighted that the molecular effect of olive polyphenols counteract others diseases via its anti-inflammatory properties, including cancer, atherosclerosis, liver steatosis and/or other liver tissue damage, obesity, type 2 diabetes, metabolic syndrome, cardiovascular diseases, amyloid and neurological diseases, between others [23,24,25,26,27,28].

Therefore, the phenolic compounds can be considered as a nutraceutical-encompassing prophylaxis and cure of diseases [29]. It is possible that a daily consumption of EVOOs will let to delay the appearance of the symptoms of aging-related pathologies leading to lower incidence of neurodegenerative disorders in the Mediterranean area [30,31]. Only a previous work reported in vitro neuroprotector effect of phenol-rich EVOO extracts from ′Cornicabra′ and ′Picual′ Spanish varieties [32]. For this scientific reason, we aimed at evaluating, for the first time, the in vitro capacity of phenolic extracts obtained from two new cultivars recently identified in Galicia, northwestern Spain (monovarietal ′Brava′ and ′Mansa′ EVOOs) to inhibit key enzymes implied in the management of neurodegenerative disorders. In addition, since the chemical profile can greatly influence the biological activities, we tried to correlate the chemical composition with the observed activities. 

## 2. Results 

### 2.1. Phenolic Profile 

**Figure 1:**
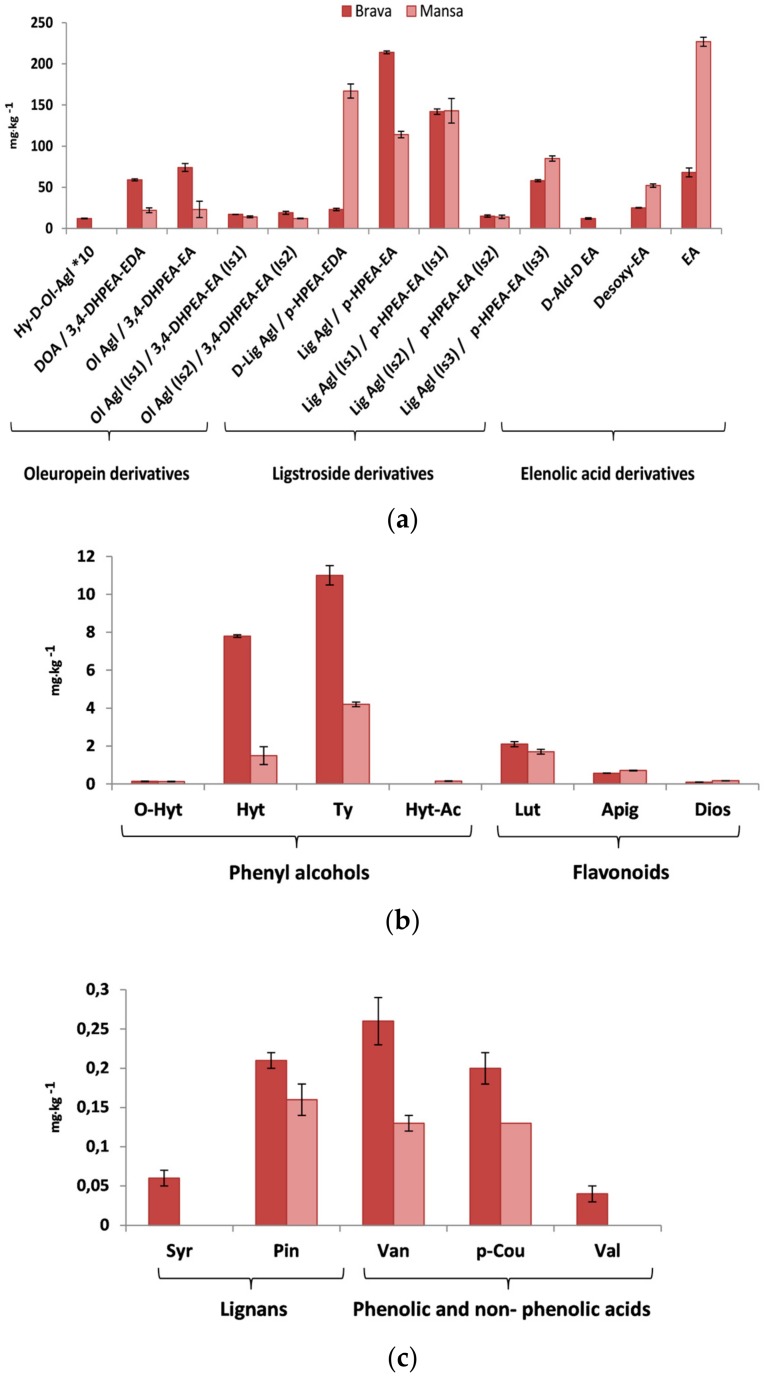
Results correspond to mean ± standard deviation (*n* = 3) of phenolic compounds (mg·kg^−1^) extracted from the evaluated EVOOs by using LC-ESI-IT-MS (**a**) secoiridoids including oleuropein derivatives, ligstroside derivatives and elenolic acid derivatives; (**b**) phenyl alcohols and flavonoids and (**c**) lignans and phenolic and non-phenolic acids. Acronyms: (**a**) Hy-d-Ol-Agl, Hydroxy decarboxymethyl oleuropein aglycone; 10-Hy-Ol-Agl, 10-Hydroxy oleuropein aglycone; DOA/3,4-DHPEA-EDA, Dialdehydic form of decarboxymethyl oleuropein aglycone/Dialdehydic form of decarboxymethyl elenolic acid linked to hydroxytyrosol; Ol Agl/3,4 DHPEA-EA, Oleuropein aglycone (main peak); Ol Agl (Is1)/3,4 DHPEA-EA (Is1), Oleuropein aglycone (Isomer 1); Ol Agl (Is2)/3,4 DHPEA-EA (Is2), Oleuropein aglycone (Isomer 2); d-Lig Agl/*p*-HPEA-EDA, Dialdehydic form of decarboxymehtyl ligstroside aglycone/Dialdehydic form of decarboxymethyl elenolic acid linked to tyrosol/Oleocanthal; Lig Agl/*p*-HPEA-EA, Ligstroside aglycone; Lig Agl (Is1)/*p*-HPEA-EA (Is1), Ligstroside aglycone (Isomer 1); Lig Agl (Is2)/*p*-HPEA-EA (Is2), Ligstroside aglycone (Isomer 2); Lig Agl (Is3)/*p*-HPEA-EA (Is3), Ligstroside aglycone (Isomer 3); d-Ald-d EA, Decarboxymethylated form of elenolic acid/Dialdehydic form of decarboxymethyl of elenolic acid; Desoxy-EA, Desoxy elenolic acid; EA, Elenolic acid. (**b**) *O*-Hyt, Oxidized hydroxytyrosol; Hyt, Hydroxytyrosol/3,4-dihydroxyphenylethanol; Ty, Tyrosol/(*p*-hydroxyphenyl)etanol; Hyt-Ac, Hydroxytyrosol acetate; Lut, Luteolin; Apig, Apigenin; Dios, Diosmetin. (**c**) Syr, Syringaresinol; Pin, Pinoresinol; AcetPin, Acetoxypinoresinol;Van, Vanillic acid; *p*-Cou, *p*-Coumaric acid; Val, Vanillin.

The phenolic profiles of monovarietal ′Brava′ and ′Mansa′ EVOOs are displayed in Figure 1. A total of 30 phenolic compounds belonging to several chemical families (such as phenolic acids, flavonoids, lignans, simple phenols or secoiridoids) characterized the complex and heterogeneous profile pattern of the tested oils. 

Secoiridoids (oleuropein and ligstroside derivatives) were the main phenolic group comprising 67–83% of the total phenolic compounds in the studied EVOOs. Quantitative differences between both olive oils can be observed when the individual secoiridoids are considered (Figure 1a). The main compounds of oleuropein derivatives were dialdehydic form of decarboxymethyl oleuropein aglycone (DOA) (3,4-DHPEA-EDA) (59 ± 1 in ′Brava′ EVOO–22 ± 3 mg·kg^−1^ in ′Mansa′ EVOO) and oleuropein aglycone (Ol Agl) (3,4 DHPEA-EA) (74 ± 5–23 ± 10 mg·kg^−1^). Among the ligstroside derivatives, ligstroside aglycone (Lig Agl) (*p*-HPEA-EA) (214 ± 2 mg·kg^−1^) was predominant in ′Brava′ oil while oleocanthal (d-Lig Agl) (*p*-HPEA-EDA) (167 ± 8 mg·kg^−1^) was in ′Mansa′ oil. In addition, elenolic acid, (EA) (nonphenolic compound) was three times higher in ′Mansa′ than in ′Brava′ oils. 

Phenyl alcohols represented the second group of the total phenols (2.5–0.66%). The main phenyl alcohols were hydroxytyrosol (Hyt) (8 ± 0.1 in ′Brava′ EVOO–1.5 ± 0.5 mg·kg^−1^ in Mansa′ EVOO) and tyrosol, (Ty) (11 ± 0.51–4.2 ± 0.12 mg·kg^−1^) (Figure 1b). In addition, luteolin (Lut) was the flavonol found in the highest concentration (2.1 ± 0.1–1.7 ± 0.1) for ′Mansa′ and ′Brava′ oils. Minority groups were represented by lignans, being pinoresinol (Pin) the main compound for both oils, and phenolic and non-phenolic acid group, being vanillic acid (Van) the main compound for target oils (Figure 1c).

### 2.2. Neuroprotective Potential 

In the current work, the activities of the phenol-rich extracts obtained from ′Brava′ and ′Mansa′ oils were investigated against these enzymes. In addition, they were compared to that of RSO, which lacks phenolic compounds. As far as we know, this is the first report in evaluating the ability to inhibit CNS-related enzymes of phenol-rich extracts from any EVOOs extracted from ′Brava′ and ′Mansa′ Galician varieties. Only one previous work reported in vitro neuroprotector effect of phenol-rich EVOO extracts from ′Cornicabra′ and ′Picual′ Spanish varieties [32]. 

**Figure 2:**
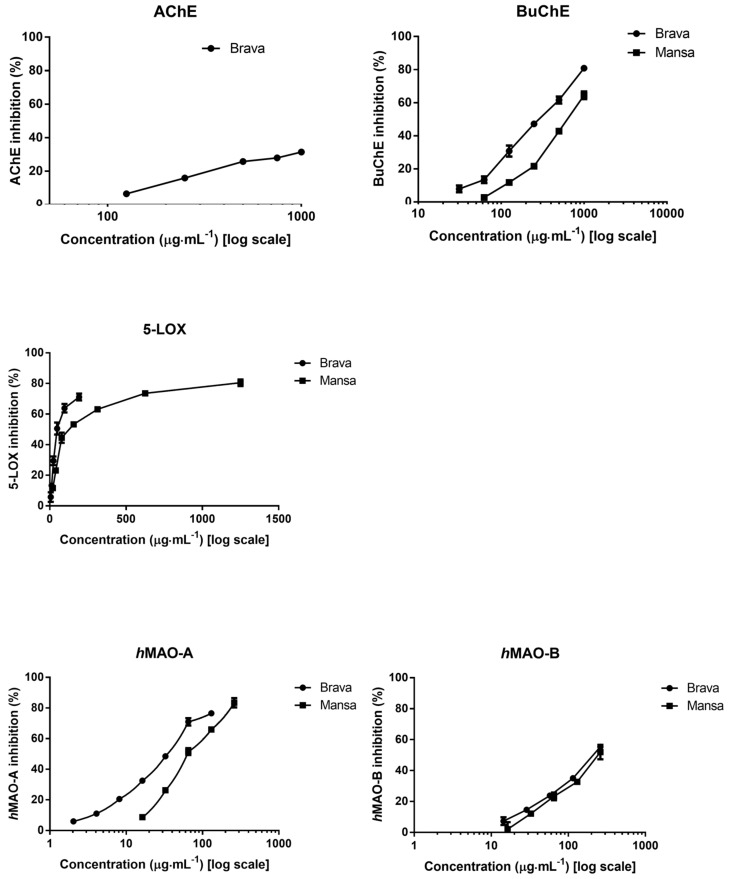
Inhibition of butyrylcholinesterase (BuChE), acetylcholinesterase (AChE), 5-lipoxygenase (5-LOX), monoamine oxidase A (*h*MAO-A) and monoamine oxidase B (*h*MAO-B) by phenol-rich extracts from ′Brava′ and ′Mansa′ EVOOs. Results are expressed as mean ± SD (μg·mL^−1^ of dry extract) of three experiments, each performed in triplicate.

***BuChE inhibition.*** As it is shown in Figure 2, ′Brava′ and ′Mansa′ extracts inhibited BuChE in a dose-dependent manner. As a measure of the inhibitory potency of the tested extracts, IC_50_ and IC_25_ values were calculated (Table 1). 

′Brava′ EVOO revealed to be more effective against BuChE (IC_50_ = 298 ± 6 μg of dry extract·mL^−1^) than ′Mansa′ EVOO (IC_50_ = 668 ± 26 μg of dry extract·mL^−1^). Different concentrations of the main reference drug, namely galanthamine, were used as positive control and ran in parallel, being the effect stronger than that obtained with any extract (IC_50_ = 7 ± 0.5 μg·mL^−1^). Similar results to those reported herein were found by Figueiredo-González et al. [32] for the phenol-rich EVOO extract from ′Cornicabra′ (IC_50_ = 249 ± 6 μg of dry extract·mL^−1^) and ′Picual′ (IC_50_ = 357 ± 38 μg of dry extract·mL^−1^) Spanish varieties. 

***AChE inhibition.*** ′Mansa′ EVOO did not displayed activity against AChE up to the highest tested concentration (1 mg of dry extract·mL^−1^). Only ′Brava′ EVOO was able to inhibit this enzyme in a concentration dependent manner (Figure 2) and in a lesser extent than against BuChE. Due to solubility issues, it was not possible to calculate the IC_50_ value, being presented the IC_25_ value (IC_25_ = 483 ± 30 μg of dry extract·mL^−1^) (Table 1). In the same way that the inhibition of the enzyme BuChE, the reference drug galantamine (IC_50_ = 2 ± 0.3 μg·mL^−1^) was more effective than that exerted by ′Brava′ EVOO. These results are in accordance with those reported by Figueiredo-González et al. [32], who found IC_25_ values of 503 ± 36 mg EVOO·mL^−1^ for ′Cornicabra′ variety. 

***LOX inhibition.*** ′Brava′ and ′Mansa′ oils were both able to inhibit this enzyme, although the effect was lower than that one showed by the positive control (quercetin, IC_50_ = 3 ± 0.2 μg·mL^−1^). The IC_50_ values were of 50 ± 8 and 124 ± 17 μg of dry extract·mL^−1^, respectively. Our results are in agreement with those recently published by Figueiredo-González et al. [32], who observed inhibitory activity against 5-LOX from 42 ± 1 and 43 ± 4 μg of dry extract·mL^−1^ for hydromethanol-based extracts obtained from ′Cornicabra′ and ′Picual′ oils, respectively. On the contrary, RSO did not displayed activity against 5-LOX up to the highest tested concentration (2 mg of dry extract·g^−1^). 

***h*MAO *inhibition*.** Data from the present study provide evidence of the concentration-dependent inhibitory effect of the tested extracts from ′Brava′ and ′Mansa′ oils on *h*MAO-A and on *h*MAO-B activities. Although the observed activity for both EVOOs against both *h*MAOs was significantly lower than that of clorgyline (*h*MAO-A, IC_50_ = 0.03 ± ˂0.01 μg·mL^−1^ and *h*MAO-B, IC_50_ = 23 ± 0.3 μg·mL^−1^), IC_50_ values were calculated (Table 1). ′Brava′ and ′Mansa′ oils displayed IC_50_ values of 35 ± 2 and 64 ± 4 μg of dry extract·mL^−1^ for *h*MAO-A and 223 ± 10 and 235 ± 16 μg of dry extract·mL^−1^ for *h*MAO-B. These results are in agreement with those reported by Figueiredo-González et al. [32] who found for ′Cornicabra′ oil IC_50_ values of 32 ± 4 and 209 ± 11 μg of dry extract·mL^−1^ for *h*MAO-A and *h*MAO-B, respectively, while ′Picual′ oil showed IC_50_ values of 43 ± 0.3 and 249 ± 15 μg of dry extract·mL^−1^ for the same enzymes. RSO had no effect on these enzymes at the highest tested concentration (686 μg of dry extract·g^−1^). 

## 3. Discussion

The reduction of acetylcholine (ACh) levels is associated with the progression of different neurological disorders including AD. Since ACh is hydrolyzed by the two major forms of cholinesterases (BuChE and AChE), their inhibition is a valuable tool to improve cognitive functions [33].

′Brava′ EVOO was the most potent against both enzymes, revealing dual ChE inhibition. There are reports suggesting that the use of compounds with dual ChE inhibition can effectively increase the efficacy of the treatment [34]. On the contrary, ′Mansa′ variety was selective BuChE inhibitor. It is could also be advantageous since the stabilization of the cognitive status of AD patients could be due to the increase in the levels of ACh, but also to the inhibition of β-amyloid plaques formation (associated to BuChE enzyme) [34,35].

The phenolic compounds of the EVOOs could be responsible of cholinesterase inhibition. In fact, RSO without any phenolic compounds did not show. Likewise, Collado-González et al. [36] did not observe inhibitory activity against either AChE or BuChE for phytoprostanes-rich extracts from ′Cornicabra′ and ′Picual′ oils. These authors also suggested that the lack of ChE activity might be due to the lack of flavonoids and other phenolic compounds from hexane extracts after being removed by a solid phase extraction (SPE) clean-up technique. 

Phenolic compounds reported in literature as owners of anticholinesterase activity are *p*-coumaric acid (*p*-Cou) and luteolin (Lut) [14,16] in addition to lignans isolated from plants such as syringaresinol (Syr) (which showed an inhibitory effect against AChE) [15], and pinoresinol (Pin) (which displayed selective inhibitory effects on BuChE, but not against AChE) [17]. The content of these compounds were higher in ′Brava′ than in ′Mansa′ olive oils, as mentioned before. However, it is important to note that the biological activity of any extract reflects not only the action of those molecules present at the highest levels, but also the possible synergistic/antagonistic interactions between compounds. 

5-LOX has been associated with AD and other aging-related events [11]. Thus, 5-LOX inhibitors could effectively reduce the inflammation associated with neurodegenerative disorders. Both oils displayed activity against 5-LOX while that RSO not inhibited this enzyme up to the highest tested concentration. This fact can possibly be due to the inhibition of 5-LOX is effective when the matrix is rich in phenolic compounds [18]. In fact, *p-*HPEA-EDA (23 ± 1–167 ± 8 mg·kg^−1^), Hyt (8 ± 0.1–2 ± 0.5 mg·kg^−1^) *p*-Cou (0.2 ± ˂0.01–0.1 ± ˂0.01 mg·kg^−1^) and Lut (2.1 ± 0.1–1.7 ± 0.1 mg·kg^−1^) in ′Brava′ and in ′Mansa′, respectively, already have been described as potential anti-inflammatories by 5-LOX inhibition. The certain synergistic effect between all olive oil phenols might also be responsible for the observed activity [13,19,20,32].

Extracts obtained from EVOOs displayed inhibitory activity against *h*MAOs. The activity observed may result from synergism and/or antagonism phenomena that occur among the several phenolic compounds and/or the presence of flavonoid compounds, such as apigenin (Apig) and Lut, [21,22] described as owners of this activity. By contrast, RSO had no effect on these enzymes, possibly due to the lack of these compounds. Our extracts displayed higher inhibitory activity towards *h*MAOs than towards cholinesterase enzymes; the last ones present the highest level of human homology. In addition, the design and synthesis of novel dual monoamine-cholinesterase inhibitors is a hot-topic in neuropharmacology research [1]. Therefore, data herein presented a great interest in the seeking of compounds to treat several neurodegenerative disorders. Our oils could be considered as an effective complementary therapy by its inhibitory effect on key enzymes linked to neurodegenerative disorders. 

**Relationship between neuroprotective potential and phenolics for EVOOs differentiation.** Phenolic compounds found in the oil extracts seem to play a role in the inhibition of neuroprotective enzymes. Thus, we have contemplated as a relevant step in our study to carry out a Pearson´s correlation test, considering individual phenolics and IC_50_ values obtained for these enzymes. In order to increase sample size, phenolic data from the studied Galician EVOO extracts as well as phenolics from Spanish ′Cornicabra′ and ′Picual′ EVOO extracts [32] were jointly considered in the statistical test. To the best of our knowledge, no studies on the possible interactions of these compounds and related neuroprotection enzymes have been reported up to date.

Table 2 shows data resulting for Pearson′s correlation test. Only data of negative correlations higher than (*r* = −0.5000) between the phenolic compounds and IC_50_ values are shown. These correlations, inversely proportional, means that EVOO extracts with high phenolic content are more active.

Regarding secoiridoids, a Pearson test showed a strongly negative correlation of total oleuropein derivatives content (*r* = −0.9069 for BuChE, *r* = −0.9263 for 5-LOX and *r* = −0.8765 for *h*-MAO-A). This fact could be mainly ascribed to the DOA (*r* = −0.8202 for BuChE, *r* = −0.7893 for 5-LOX and *r* = −0.7757 for *h*MAO-A) and in less extension to Olg Agl (*r* = −0.6791 for BuChE, *r* = −0.7845 for 5-LOX and *r* = −0.6947 for *h*MAO-A). On the contrary, for *h*MAO-B the strongly negative correlation between IC_50_ value for this enzyme and total ligstroside derivatives content (Lig Agl , *r* = −0.7731) was observed. On the other hand, lignans show strongly negative correlations with IC_50_ values calculated for BuChE (*r* = −0.7006), 5-LOX (*r* = 0.7220), *h*MAO-A (*r* = −0.6611) and *h*MAO-B (*r* = −0.6570) enzymes being Syr and Pin responsible (except for Pin in the case of *h*MAO-B inhibition). The different levels of correlation found point to the influence, at least partial, of phenolics from olive oil and/or synergistic/antagonistic interactions between them as responsible of tested activities. 

As the interesting correlations are negative, linking high phenolic concentrations with low levels of IC_50_, for the following multivariate statistics (*viz* cluster analysis, PLS and discriminant analysis), IC_50_ values were sign-changed. In this sense, it is easy to see positive correlations in bidimensional spaces since the variables will occupy a close position.

**Figure 3:**
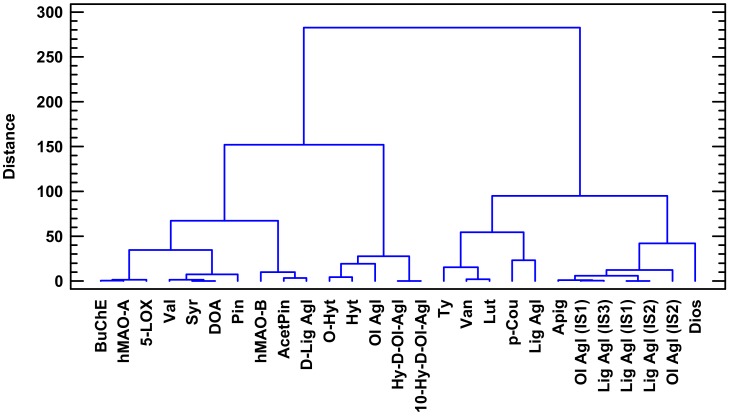
Dendrogram grouping variables according to the squared Euclidean similitude distance by Ward′s method. Enzymatic variables of BuChE, *h*MAO-A and 5-LOX were highly correlated with phenolic variables of Val, Syr, DOA and Pin. All of them were closely connected to *h*MAO-B, which was highly correlated with AcetPin and d-Lig Agl. The rest of phenolic compounds were separated at linkage distances higher than 150. Variables abbreviations are described in Figure 1.

Choosing a relatively large and safe cutting value at the linkage distance of 200 in the dendrogram (Figure 3), the variables of IC_50_ and phenolics could be divided into two main clusters or groups. The first group cluster on the left could be further divided at the linkage distance of 100 into 2 subgroups. The first subgroup on the left contains all IC_50_ values connected to a few phenolics concentration values. If we further split the first subgroup on the left at a distance of 50, the IC_50_ values of BuChE, *h*MAO-A and 5-LOX enzymes are clustered at a very small distance with Val, Syr, DOA and Pin. In the same way, *h*MAO-B is associated with acetoxypinoresinol (AcetPin) and d-Lig Agl phenolics. 

**Figure 4:**
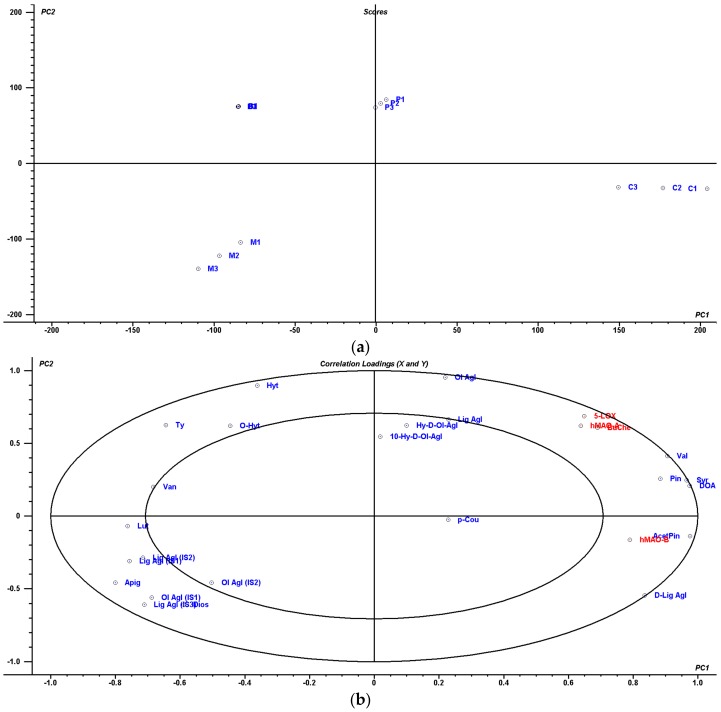
Two-dimensional PLS2: (**a**) scores plot for ′Mansa′ (M), ′Cornicabra′ (C), ′Brava′ (B) and ′Picual′ (P) EVOOs, together with (**b**) correlations between the loadings of X (phenolic profiles in blue) and Y variables (IC_50_ values in red). In the same way as for Figure 4, enzymatic variables of BuChE, *h*MAO-A and 5-LOX were highly correlated with phenolic variables of Val, Syr, DOA and Pin. All of them were closely connected to *h*MAO-B, which was highly correlated with AcetPin and d-Lig Agl. The rest of phenolic compounds were separated at higher similitude distances. Variables abbreviations are described in Figure 1.

A PLS2 was also used to correlate phenolic profiles with IC_50_ enzymatic data. PLS2 modelling provided a two-factor model explaining 98% of the variance in X (phenolic profiles) and 51% of that in Y (IC_50_ enzymatic data) (Figure 4). The ensuing model was evaluated via the root mean square error for predictions (RMSEP), which was calculated to be lower than 10 for IC_50_ values. The scores plot in Figure 4a shows how the four target EVOOs can be separated in different quadrants. In this sense, PC1 can separate EVOOs in the following order: ′Cornicabra′ >> ′Picual′ > ′Brava′ = ′Mansa′. To further separate ′Brava′ and ′Mansa′ EVOOs it is necessary PC2; with its help ′Brava′ > ′Mansa′. The central ellipsoid in Figure 4b indicates that all variables inside the ellipsoid were correlated amongst them (*r* > 0.700) and explained variation almost the EVOOs samples. In the same way as for Figure 3, enzymatic variables of BuChE, *h*MAO-A and 5-LOX were highly correlated with phenolic variables of Val, Syr, DOA and Pin. All of them were closely connected to *h*MAO-B, which was highly correlated with AcetPin and d-Lig Agl. All these four enzymatic IC_50_ values and six phenolic compounds have a higher weight in PC1 to separate ′Cornicabra′ EVOOs from the rest. To separate the rest of EVOOs, PC2 is very useful, connecting ′Picual′ with mainly Lig Agl and Ol Agl, which are in the same direction of the bidimensional space. ′Brava′ is associated with the phenolics in the same up-left quadrant, and ′Mansa′ with the phenolics in the down-left quadrant. All this supports the idea of ′Cornicabra′ being the best of the EVOOs, followed by ′Picual′ and then ′Mansa′ and ′Brava′, according to their effects on the inhibition of enzymes related with neurodegenerative processes, but also all these EVOOs can be separated by a specific profile of phenolics.

**Figure 5:**
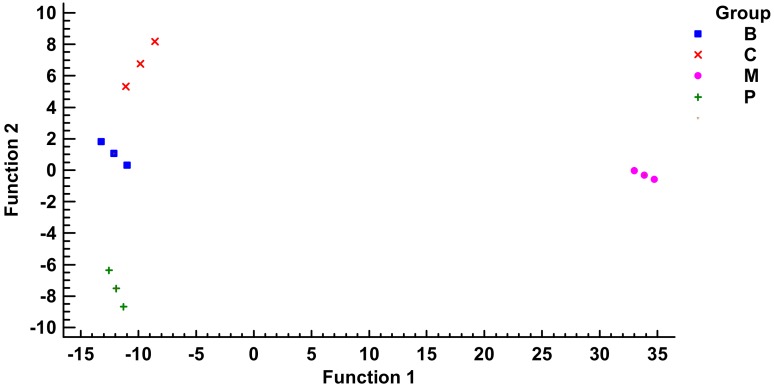
Discriminant biplot for the classification variables of IC_50_ values (BuChE, 5-LOX, *h*MAO-A and *h*MAO-B). Note that linear discriminant function 1 accounts for the 93% of variance and clearly separates ′Mansa′ (M) EVOO from the rest, mainly because of the weight of BuChE but also *h*MAO-A, whereas the discriminant function 2 with a 6% of the variance allows the discrimination between ′Cornicabra′ (C), ′Brava′ (B) and ′Picual′ (P) EVOOs with the weights of *h*MAO-B and 5-LOX.

Since the IC_50_ values can be used alone to separate oils, a discriminant analysis was performed using only this kind of data (Figure 5). Note that linear discriminant function 1 accounts for the 93% of variance and clearly separates ′Mansa′ EVOO from the rest, mainly because of the weight of BuChE but also *h*MAO-A, whereas the discriminant function 2 with a 6% of the variance allows the discrimination between ′Cornicabra′, ′Brava′ and ′Picual′ EVOOs with the weights of *h*MAO-B and 5-LOX. However, further investigation is needed to determine the individual phenol components present in olive oils may be implied for improvements in neurodegenerative disorders, by regulating enzymes inhibitory activities, as well as the possible synergistic/antagonistic phenomena amongst them. 

## 4. Materials and Methods

### 4.1. Chemicals and Standards

Galantamine, AChE (from electric eel), acetylthiocholine iodide (ATCI), BuChE (from equine serum), *S*-butyrylthiocholine iodide (BTCI), bovine serum albumin (BSA), 5,5-dithiobis (2-nitrobenzoic acid) (DTNB), Trizma^®^hydrochloride (Tris–HCl), *N*-Methyl-*N*-propargyl-3-(2,4-dichlorophenoxy)propylamine hydrochloride or clorgyline, kynuramine dihydrobromide crystalline, monoamine oxidase A and B (human recombinant, expressed in baculovirus infected BTI insect cells), lipoxydase (from glycine max), linoleic acid, quercetin, sodium hydroxide, potassium dihydrogen phosphate and sodium chloride were obtained from Sigma-Aldrich (St. Louis, MO, USA). Magnesium chloride hexahydrate (MgCl_2_·6H_2_O) was purchased from Merck (Darmstadt, Germany).

### 4.2. Extra-Virgin Olive Oil Samples

′Brava′ and ′Mansa′ are two ancient cultivars recently identified from northwestern Spain [37]. Olives were carefully harvested in November 2016 in a cultivation area located between two municipalities, Ribas do Sil (42°27′59.8′′ N 7°17′15.8″ W) and Quiroga (42°29′04.8′′ N 7°12′33.4′′ W), placed at Lugo province (NW Spain). Olive fruits were grown under organic agricultural practices. The trees were in dry condition, and subscriber and sanitary techniques suitable for cultivation in the studied area controlled their nutritional and sanitary status.

Two monovarietal olive oils from ′Brava′ and ′Mansa′ cultivars, separately, were obtained under optimal conditions with an OLIOMIO 50 (Oliomio, Conegliano, Italy), equipped with a knife crusher, a horizontal malaxator and a two-phase decanter. The malaxation trials were carried out for 45 minutes, and a temperature of 18 ± 2 °C for both varieties on two consecutive days. Once in the laboratory, three different bottles of 500 mL from each variety were pooled and homogenized to obtain a final representative sample prior to analysis. The samples were kept at a constant temperature of 4 °C until analysis using amber bottles without headspace.

Both monovarietal oils were classified as extra virgin, considering that their quality indices (physicochemical and sensory quality parameters) fell within the ranges established by legislation [38].

### 4.3. Phenolic Compounds Analysis

Phenolic compounds were extracted, by duplicate, from EVOOs according to the methodology proposed by Bajoub et al. [39]. Identification and quantification of polyphenols were performed according to the LC-ESI-IT-MS procedures described by Figueiredo-González et al. [32].

### 4.4. In Vitro Enzyme Inhibition. Potential Neuroprotective Activity

In parallel with rich-phenol extracts from EVOOs, the activity of a refined sunflower oil (RSO) was also assessed. RSO does not contain phenolic compounds and can be considered as a blank matrix to evaluate the effect of bioactive compounds.

*Cholinesterases.* Inhibition of AChE and BuChE was assessed according to a previously described methodology [40]. Briefly, the extract dissolved in buffer A (50 mM Tris-HCl, pH 8) or only buffer A without extract (negative control) was added to each well, together with ATCI or BTCI. Then, DTNB and buffer B (50 mM Tris-HCl, with 0.1% BSA, pH 8) were added. The absorbance was measured at 405 nm in a LT-5000 MS ELISA READER (Labtech.com, Palaiseau, France). The rates of reactions were calculated after addition of AChE (0.44 U mL^−1^ in buffer B) or of BuChE (0.10 U mL^−1^ in buffer B). Galantamine was used as positive control. 

*Lipoxygenase.* The inhibitory effect on 5-LOX was assessed in 96-well plates, using a previously documented procedure [41]. Briefly, 20 μL of extracts, 200 μl of phosphate buffer (pH 9) and 20 μL of 5-LOX (100 U) were added to each well. After 5 min of pre-incubation at room temperature, the reaction was started by addition of 20 μL of linoleic acid (4.18 mM in ethanol). The reaction time was 3 min. The absorbance was measured at 234 nm in a FLUOstar Omega Microplate Reader—BMG LABTECH (Weston, FL, USA). Quercetin was used as positive control.

*Monoamine oxidases*. Inhibition of *h*MAO-A and *h*MAO-B was assessed according to a methodology that has been described before by [1]. Concisely, a mixture of kynuramine and the extract dissolved in phosphate buffer (pH 7.4) or just buffer (negative control) was incubated for 10 min, at 37 °C. The reaction was initiated by adding of *h*MAOs (17 U mL^−1^) and the mixture was further incubated at 37 °C for 70 min. Afterwards, the reaction was stopped by the addition of NaOH 2 N and the absorbance was measured at 314 nm using a spectrophotometer (Beckman Coulter, Brea, CA, DU 730, USA, Life Science UV/Vis spectrophotometer). Clorgyline was tested as positive control. 

### 4.5. Multivariate Statistical Analysis

The IC_50_ values were calculated from three independent assays, each of them performed in triplicate, and all results are presented as mean values. Values obtained were compared using unpaired *t*-test (GraphPad Prism 6 Software, Inc., San Diego, CA, USA). Differences at *p* < 0.05 were considered statistically significant, as well as Pearson partial correlations between both sets of variables: IC_50_ and phenolic concentrations.

A cluster analysis to group variables, both IC_50_ and phenolic concentrations, based on the squared Euclidean similitude distance by Ward′s method [42] together with discriminant analyses to separate EVOOs by means of IC_50_ values were also performed at a *p* < 0.05 with the statistical software package Statgraphics Centurion XVI from StatPoint Technologies Inc. (Warrenton, VA, USA). 

PLS is a method for relating two data matrices, IC_50_ (Y matrix) and phenolic concentrations (X matrix), through a linear multivariate model, performed with The Unscrambler soft from CAMO. The idea is to relate a matrix of responses Y to the predictor variables of matrix X. To this end, matrix X is successively deflated; PLS seeks the directions in the X and Y-spaces corresponding to the maximum covariance. In this way, PLS forms principal components as linear combinations of the original variables, which are then related to the samples scores via a linear model. By plotting the principal components, one can view interrelationships between different variables sets (IC_50_ and phenolic concentrations), and detect and interpret sample patterns, groupings, similarities or differences.

## 5. Conclusions

This work successfully demonstrates, for the first time, that rich-phenolic extracts obtained from Galician EVOOs could act as multi-target ligands directly inhibiting CNS-related enzymes. Although both EVOOs exhibited a weaker activity than that of the positive controls, they were able to inhibit simultaneously BuChE, 5-LOX, *h*MAO-A and *h*MAO-B, in a dose-dependent manner. Only ′Brava′ oil also showed a dual inhibition against both cholinesterase enzymes, which could imply increase the efficacy of the treatment of several disorders affecting the CNS. Therefore, the oils could be potential candidates in developing medicinal preparations and nutraceutical or functional foods for these disorders. 

According to Pearson′s correlation test, the phenolic compounds with a greater influence on BuChE, 5-LOX and *h*MAOs inhibition were some secoiridoids derivatives, lignans and vanillic acid. Although the cell-free model assays cannot be simply extrapolated to in vivo, they must be interpreted as easy and fast tools for a first approach. Thus, this work provides an approximation more on the nutraceutical properties of the EVOO phenolics, a valuable potential yet to be explored. In this regard, clinical trials are needed to provide a broader insight on the structure–activity relationship of olive oil polyphenols.

## Figures and Tables

**Table 1:** Effect of the extra-virgin olive oil (EVOOs) from ′Brava′ and ′Mansa′ varieties and the positive controls against enzymes involved in neurodegenerative disorders.

	EVOOs		Positive Controls		
	′Brava′ μg Dry Extract·mL^−1^	′Mansa′ μg Dry Extract·mL^−1^	Galanthamine μg·mL^−1^	Quercetin μg·mL^−1^	Clorgyline μg·mL^−1^
Neuroprotection					
BuChE ^1^	298 ± 6 ^a^	668 ± 26 ^b^	7 ± 0.5		
AChE ^2,3^	483 ± 30 ^a^	--	2 ± 0.3		
5-LOX ^1^	50 ± 8 ^a^	124 ± 17 ^b^		3 ± 0.2	
*h*MAO-A ^1^	35 ± 2 ^a^	64 ± 4 ^b^			0.03 ± ˂0.01
*h*MAO-B ^1^	223 ± 10 ^a^	235 ± 16 ^a^			23 ± 0.3

^1^ IC_50_ values for EVOOs and positive controls; ^2^ IC_25_ values for EVOO; ^3^ IC_50_ values for positive control; -- Inhibition effect not found. Different letters indicate significant differences (*p* < 0.05) between values expressed as μg of dry extract·mL^−1^ for the EVOOs.

**Table 2:** Pearson correlation coefficients between the phenolic compounds in ′Brava′, ′Mansa′, ′Cornicabra′ and ′Picual′ olive oils (3 samples per oil and *n* = 12, *p* < 0.05) and the IC_50_ inhibitory activities for BuChE, 5-LOX and *h*MAOs in the EVOOs. IC_25_ inhibitory activity for AChE was not considered because of lack of data for ′Mansa′ and ′Picual′; the same was taken into account in Figure 3, Figure 4 and Figure 5 with multivariate statistics.

Phenolic Compounds			BuChE	5-LOX	*h*MAO-A	*h*MAO-B
Secoiridois	Oleuropein derivatives	DOA/3,4-DHPEA-EDA	−0.8202	−0.7893	−0.7757	−0.7637
		Ol Agl/3,4 DHPEA-EA	−0.6791	−0.7845	−0.6947	
	*Total*		−0.9069	−0.9263	−0.8765	−0.5123
	Ligstroside derivatives	Lig Agl/*p*-HPEA-EA	−0.7231	−0.5837	−0.6705	−0.7731
	*Total*					−0.7583
	Total		−0.5804		−0.5351	−0.9377
Lignans		Syr	−0.8362	−0.8168	−0.7996	−0.7489
		Pin	−0.6418	−0.7162	−0.6069	
	Total		−0.7006	−0.7220	−0.6611	−0.6570
Phenolic acids		*p*-cou				−0.6388
		Val	−0.8833	−0.8870	−0.8544	−0.6545
	Total					

DOA/3,4-DHPEA-EDA, Dialdehydic form of decarboxymethyl oleuropein aglycone/Dialdehydic form of decarboxymethyl elenolic acid linked to hydroxytyrosol; Ol Agl/3,4 DHPEA-EA, Oleuropein aglycone; Lig Agl/*p*-HPEA-EA, Ligstroside aglycone; *O*-Hyt, Oxidized hydroxytyrosol; Hyt, Hydroxytyrosol/3,4-dihydroxyphenylethanol; Ty, Tyrosol/(*p-*hydroxyphenyl)etanol; Apig, Apigenin; Dios, Diosmetin; Syr, Syringaresinol; Pin, Pinoresinol; *p*-Cou, *p-*Coumaric acid; Val, Vanillin.
